# Supplementary material for: Effects of Virtual Reality Exercise Program on Blood Glucose, Body Composition, and Exercise Immersion in Patients with Type 2 Diabetes
Source: Int J Environ Res Public Health. 2023 Feb 26;20(5):4178. doi: 10.3390/ijerph20054178 (PMC10002193; doi:10.3390/ijerph20054178)
Supplement: Supplementary file 1 [file ijerph-20-04178-s001.zip › ijerph-2211067-supplementary.pdf]

360VRFit Cycle2		
You can download it from the link below.<br>https://play.google.com/store/apps/details?id=vrfit.mtome.com.cycle2_360	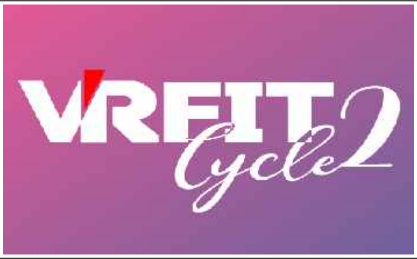	It's saved on your phone.
Register ID and password	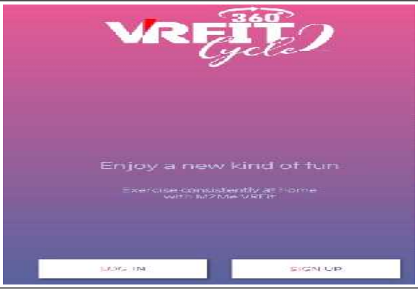	Click the SIGN UP part, register your ID and password, and check the registered email to register.
Turn on “on” by turning the visible part on the side of the sensor upward.	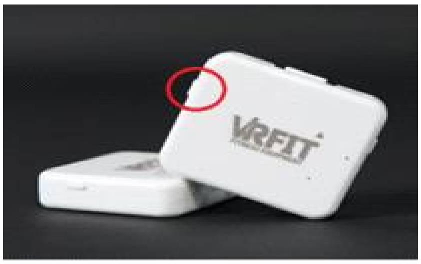	
Attach the sensor to the pedals of your bike	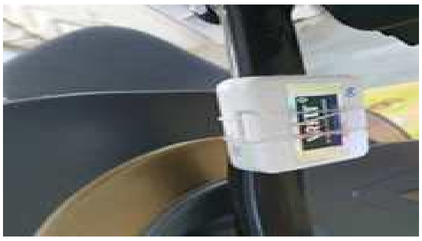	Attach the sensor to the pedals of your bike
Start exercising after registering the sensor	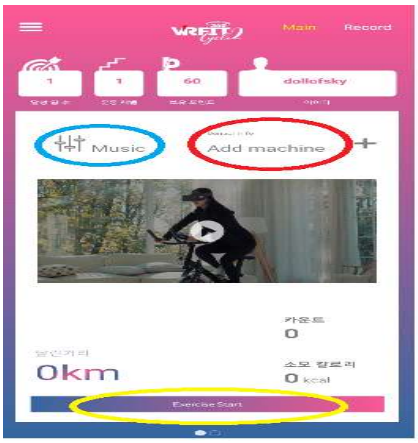	○ Click this part to set the sensor<br>○ Tap here to set pre-workout music<br>○ Click here to start the exercise.
If you put the set mobile phone into the HMD and start exercising, it will look like this screen.	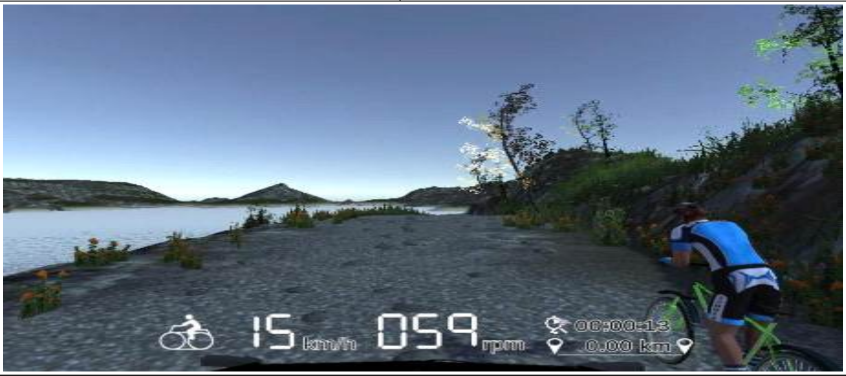	
